# Targeting Pathways Implicated in Cholesterol Metabolism for Novel Cancer Therapy

**DOI:** 10.3390/cancers18030428

**Published:** 2026-01-28

**Authors:** Yi Zhou, Vishakha Sharma, Xiaoyu Li, Rajeev K. Singla, Ankush Kumar, Ashishkumar Kyada, Suhas Ballal, Deepak Nathiya, Apurva Koul, Mohammad Khalid, Monica Gulati, Sandeep Arora, Tapan Behl, Joachim Kavalakatt, Bairong Shen, Anupam Bishayee

**Affiliations:** 1 Department of Pharmacy and Institutes for Systems Genetics, Center for High Altitude Medicine, Frontiers Science Center for Disease-Related Molecular Network, West China Hospital, Sichuan University, Chengdu 610041, China; 2 Sichuan Academy of Chinese Medicine Sciences, Chengdu 610041, China; 3 Amity School of Pharmaceutical Sciences, Amity University, Mohali 140 306, Punjab, India; 4 School of Pharmaceutical Sciences, Lovely Professional University, Phagwara 144 411, Punjab, India; 5 Marwadi University Research Center, Department of Pharmacy, Faculty of Health Sciences, Marwadi University, Rajkot 360 003, Gujarat, India; 6 Department of Chemistry and Biochemistry, School of Sciences, JAIN (Deemed to be University), Bangalore 560 041, Karnataka, India; 7 Department of Pharmacy Practice, NIMS Institute of Pharmacy, NIMS University Rajasthan, Jaipur 303 121, Rajasthan, India; 8 Chandigarh Pharmacy College, Chandigarh Group of Colleges, Mohali 140 307, Punjab, India; 9 Department of Pharmacognosy, College of Pharmacy, Prince Sattam Bin Abdulaziz University, Alkharj 11942, Saudi Arabia; 10 Department of Pharmacology, College of Osteopathic Medicine, Lake Erie College of Osteopathic Medicine, Bradenton, FL 34211, USA

**Keywords:** cholesterol metabolism, cancer, signaling pathway, molecular targets, biosynthesis

## Abstract

Cholesterol acts as a metabolic cue that reshapes diverse signaling networks, including hedgehog and several sterol-regulated pathways orchestrated by key proteins, including sterol regulatory element-binding protein 2 (SREBP2), sterol O-acyltransferase 1 (SOAT1), Niemann–Pick type C1 (NPC1), and proprotein convertase subtilisin/kexin type 9 (PCSK9). Research over the past decade has highlighted cholesterol metabolism as a key modulator of cancer development and a promising therapeutic target. By integrating mechanistic and translational evidence, this review seeks to clarify how cholesterol metabolism interfaces with oncogenic signaling and set directions for future investigation. Accumulating preclinical and clinical data suggest that dysregulated cholesterol levels, often associated with high-fat diets, may contribute to tumorigenesis and malignant transformation. Implicated pathways, such as SREBP, NPC1, PCSK9, and SOAT1, orchestrate various processes of lipid metabolism, including cholesterol synthesis, esterification, receptor degradation, and transport, that harbor a tumorigenic environment and promote oncogenic processes. Additionally, these enzymes and corresponding pathways provide a promising direction for developing metabolism-oriented anticancer strategies. Cholesterol metabolism dysregulation serves as a major avenue for cancer signaling and growth, but studies also highlight key molecular mechanisms and targets for future treatments. Future studies should focus on expanding studies into further cancer types, investigating combination therapies, and developing novel inhibitors of key molecular targets.

## Simple Summary

Cholesterol is known for its function in cell metabolism and lipid transport, but it also serves as a powerful signal that can influence cancer growth. This review seeks to understand the various pathways and mechanisms through which the dysregulation of cholesterol metabolism is implicated in cancer growth and signaling, focusing on key molecular target proteins involved in cholesterol synthesis, transport, and metabolism. Additionally, the review further assesses the potential for targeting these key pathways and proteins as a future strategy of anticancer treatment and therapy.

## 1. Introduction

Cholesterol is a vital component in building cellular structures and regulates various physiological processes [1,2]. Cholesterol sustains mammalian cellular function by stabilizing membrane organization and acting as a metabolic precursor for signaling molecules such as bile acids and steroid hormones [3,4]. Excessive accumulation of cholesterol in the bloodstream predisposes individuals to atherosclerosis and related cardiovascular disorders [5]. Composed largely of cholesteryl esters at their core, low-density lipoprotein (LDL) and high-density lipoprotein (HDL) facilitate the bidirectional transfer of cholesterol between the liver and peripheral cells [6]. LDL serves as one of the key biochemical mediators in increasing the deposition of cholesterol in arteries, a complex, multifactorial inflammatory process that ultimately drives the pathologies of heart disease and atherosclerosis [7,8]. Beyond its role in cardiovascular disease, LDL-mediated cholesterol transport is recognized as a critical metabolic adaptation in cancer, where rapidly proliferating tumor cells frequently reprogram cholesterol uptake through enhanced LDL receptor-mediated internalization. This elevated intracellular cholesterol pool supports accelerated membrane biogenesis, lipid raft formation, and oncogenic signaling, while reducing the energy cost in de novo cholesterol synthesis, thereby conferring a growth advantage in the tumor microenvironment [9,10].

Both clinical and experimental observations suggest that alterations in cholesterol metabolism play a significant role in cancer development [11,12,13,14,15]. Growing research indicates that cholesterol metabolism intricately shapes diverse aspects of tumor behavior by reprogramming oncogenic pathways, influencing ferroptosis, and altering the tumor microenvironment [16,17]. Preclinical data suggest that disruption of cholesterol metabolic pathways, including synthesis and uptake, restrains oncogenic transformation and tumor proliferation [18]. Despite increasing interest, the current state of literature broadly discusses cholesterol metabolism in cancer without thoroughly examining the specific signaling pathways and regulatory proteins that orchestrate these processes [19,20]. This review focuses on unraveling the central regulatory roles of key molecular pathways in cholesterol metabolism, with particular attention to SREBP2, SOAT1, NPC1, and PCSK9. These pathways pathologically impact lipid raft formation, membrane receptor signaling, and immune cell function, which contribute to tumor growth and metastasis. By synthesizing recent preclinical and clinical findings, we highlight the therapeutic potential of targeting these pathways for cancer therapy.

## 2. Literature Search Strategy

### 2.1. Database and Time Frame

We searched PubMed and the Web of Science Core Collection from database inception to September 2025 to identify studies on cholesterol metabolism pathways as therapeutic targets in cancer.

### 2.2. Search Concepts and Keywords

Search terms were developed using controlled vocabulary, such as Medical Subject Headings (MeSHs) and free-text keywords. For cholesterol metabolism and lipid homeostasis, we used the following keywords: “cholesterol metabolism,” “cholesterol biosynthesis,” “mevalonate pathway,” “cholesterol efflux,” “cholesterol transport,” “lipid raft,” and “lipoprotein*”. The cancer-, treatment-, and target-related keywords were “cancer”, “tumor/tumour”, “neoplasm”, “malignan”, “carcinoma”, “therapy”, “therapeutic target”, “SREBP2”, “SOAT1/ACAT1”, “NPC1”, “PCSK9”, “LXR”, “LDLR”, “HMGCR”, “statin”, “itraconazole”, “U18666A”, “SOAT inhibitor”, and “PCSK9 inhibitor”.

### 2.3. Eligibility Criteria

We included original studies (preclinical and clinical) and high-quality reviews that examined cholesterol metabolic regulation in cancer biology or therapy, with priority given to evidence involving SREBP2, SOAT1, NPC1, and PCSK9 and related effectors. We excluded conference abstracts without full text, non-cancer studies not informing oncologic mechanisms, and articles lacking relevance to cholesterol pathway modulation or cancer outcomes.

### 2.4. Study Selection and Synthesis

The titles and abstracts of publications were screened first, followed by full-text assessment. Reference lists of key articles were hand-searched to capture additional relevant studies. Evidence was synthesized narratively, emphasizing mechanistic links and translational/therapeutic implications.

## 3. Molecular Regulation of Cholesterol Metabolism: Key Pathways and Effectors

### 3.1. Cholesterol Biosynthesis Pathway

**Figure 1:**
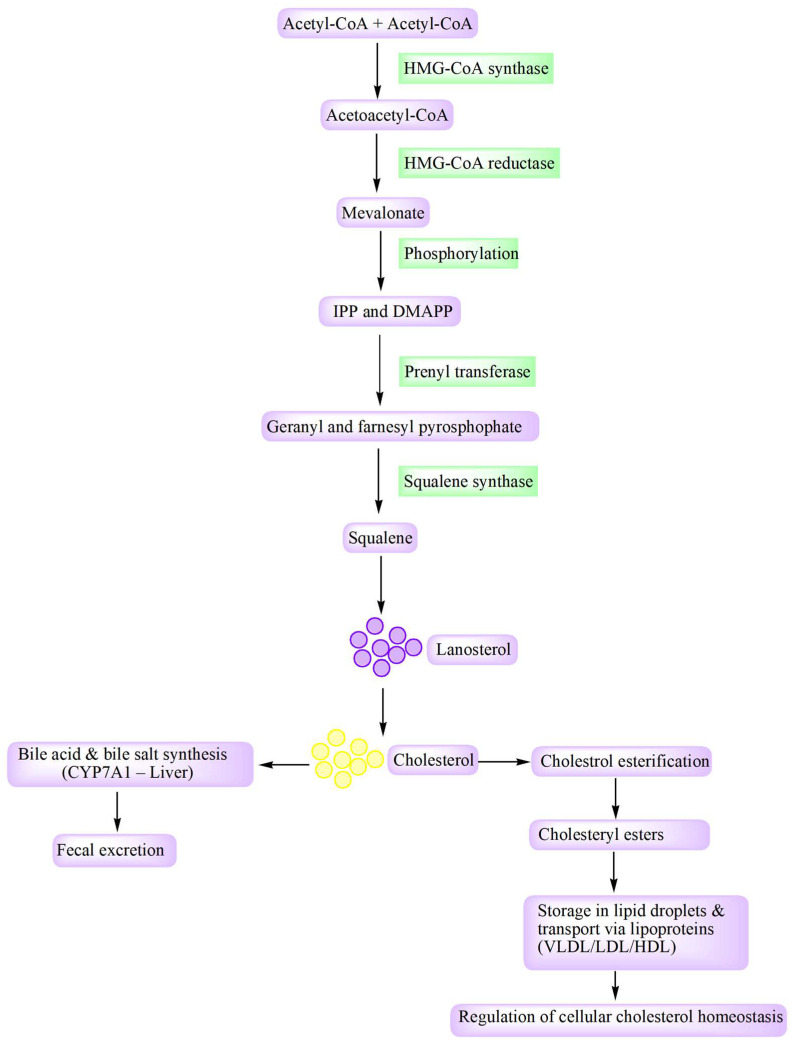
Cholesterol biosynthesis and key downstream fates. Acetyl-CoA is converted to mevalonate via HMG-CoA synthase and the rate-limiting enzyme HMG-CoA reductase, then to IPP/DMAP and downstream isoprenoids (geranyl and farnesyl pyrophosphate), which form squalene and lanosterol and ultimately cholesterol. Cholesterol is subsequently converted to bile acids/bile salts in the liver (CYP7A1) for fecal excretion or esterified to cholesteryl esters for storage and transport in lipoproteins (VLDL/LDL/HDL), supporting cellular cholesterol homeostasis. Abbreviations: Acetyl-CoA, acetyl coenzyme A; DMAPP, dimethylallyl pyrophosphate; HDL, high-density lipoprotein; HMG-CoA, 3-hydroxy-3-methylglutaryl coenzyme A; IPP, isopentyl pyrophosphate; LDL, low-density lipoprotein; VLDL, very low-density lipoprotein.

Cholesterol serves as a vital lipid constituent within the membranes of mammalian cells [21,22,23]. The cellular regulation of cholesterol levels involves a tightly coordinated network overseeing its synthesis, import, export, conversion, esterification, and movement across intracellular compartments [24,25,26]. Around 80% of the total cholesterol required by humans is typically biosynthesized within the body, while the remaining 20% is obtained from dietary sources [27,28]. The biosynthesis pathway of cholesterol is depicted in Figure 1, where we can observe that the cholesterol biosynthesis initiator is acetyl coenzyme A (acetyl-CoA) [29]. Cholesterol biosynthesis begins with the condensation of two acetyl-CoA molecules catalyzed by 3-hydroxy-3-methylglutaryl coenzyme A (HMG-CoA) synthase, producing acetoacetyl-CoA. Reduction of this intermediate by HMG-CoA reductase gives rise to mevalonate, which undergoes sequential phosphorylation to generate isopentyl and dimethylallyl pyrophosphate. These activated isoprenoids are subsequently linked by prenyl transferase to form geranyl and farnesyl pyrophosphate intermediates. Further, squalene synthase converts these products into squalene (intermediate), a triterpene composed of six isoprene units. Through epoxidation of squalene, it gets converted to lanosterol, which further leads to the synthesis of cholesterol and other sterols [30,31].

### 3.2. Liver X Receptors and Cholesterol Efflux: Central Mechanisms Governing Lipid Homeostasis

**Figure 2:**
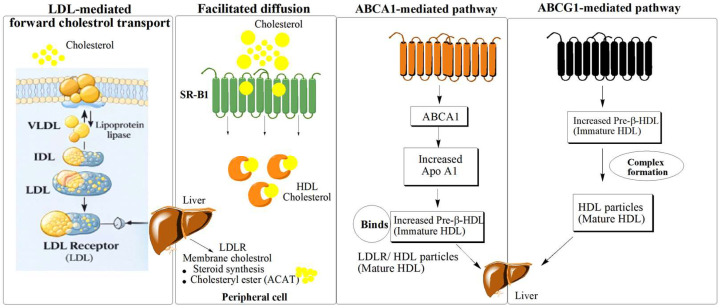
Major pathways of cellular cholesterol efflux and the dual role of SR-B1 in HDL flux. Cellular cholesterol efflux to high-density lipoprotein occurs via aqueous diffusion, ATP-binding cassette transporter A1-mediated export to lipid-poor apolipoprotein A-I to generate nascent (pre-β) high-density lipoprotein, ATP-binding cassette transporter G1-mediated export to mature high-density lipoprotein, and scavenger receptor class B type 1-facilitated diffusion. Notably, scavenger receptor class B type 1 is context-dependent and can mediate bidirectional cholesterol movement, promoting cholesterol efflux from peripheral cells to high-density lipoprotein while also enabling selective uptake of high-density lipoprotein-derived cholesteryl esters in the liver and steroidogenic tissues, thereby supporting reverse cholesterol transport and hepatic clearance. Abbreviations: ABCA1, ATP-binding cassette transporter A1; ABCG1, ATP-binding cassette subfamily G1; HDL, high-density lipoprotein; LDL, low-density lipoprotein; SR-B1, scavenger receptor class B member 1.

Cholesterol efflux represents a key regulatory pathway that expels excess intracellular cholesterol, safeguarding lipid homeostasis and ensuring proper cellular physiology [12]. Cholesterol efflux prevents cholesterol accumulation in arteries, working against plaque formation and the development of atherosclerosis [32]. During cholesterol efflux, an acceptor molecule is required to transport the cholesterol [33]. HDL acts as an acceptor of cholesterol, transporting the compound to the liver through four mechanisms [34,35]: aqueous diffusion, the ATP-binding cassette transporter A1 (ABCA1)-mediated pathway, the ATP-binding cassette transporter G1 (ABCG1)-mediated pathway, and facilitated diffusion (Figure 2). In aqueous diffusion, the cholesterol diffuses from the plasma membrane to HDL through the aqueous medium [36,37]. Lecithin–cholesterol acyltransferase (LCAT) functions as a key enzyme in reverse cholesterol transport (RCT), mediating the transfer and esterification of free cholesterol within HDL particles. After this chemical complex formation, cholesteryl esters are transferred to different lipoproteins of the body in the serum. This step is part of a physiological process, RCT. In RCT, excess cholesterol is cleared from peripheral cells and directed to the liver for biliary excretion. Dysregulation of this pathway causes cholesterol retention within cells, a condition linked to increased cancer cell proliferation, resistance to apoptosis, and metastatic progression [38]. Facilitated diffusion of cholesterol is carried out by regulation of a homo-oligomeric glycoprotein known as scavenger receptor class B, type 1 (SR-B1) [21], which is a type of HDL receptor that transfers the HDL-cholesterol to the liver through a hydrophobic channel of the SR-B1 receptor. Larger HDL particles bind tightly with SR-B1 than small ones and promote more free cholesterol efflux because even after saturation, SR-B1 receptor continue to reorganize cholesterol into the cell.

ABCA1 and aqueous diffusion are two major pathways to efflux 70% of total cholesterol prominently from the cell membrane of macrophages to pre-formed HDL particles [39], while the rest of the removal is carried out by other proteins [40]. ABCA1 is a gene that is known for the presence of four domains, two of which are nucleotide-binding domains (NBD), while the other two are six-helix transmembrane domains (TMD) [41]. Chen and the team indicated nine models to clarify the possible mechanism of cholesterol efflux by the ABCA1 pathway, and we discuss two models here as a mechanism elucidation [42]. The earliest conceptual framework describing this process is the molecular efflux, often referred to as the sequential model. This model states that the ABCA1 gene enhances the production of lipid-enriched microdomains, which further provides an active site for apo A1 [43]. The lipidation process of apo A1 gives rise to phospholipid-rich pre-β1 HDL particles that efficiently mobilize cholesterol from various intracellular and membrane regions [44]. The membrane solubilization model proposes that α-helical domains of apo A1 insert into cellular lipid bilayers, triggering apo A1-lipid clustering and partial membrane dissolution, which ultimately yields discoidal HDL particles [45]. The process culminates in the coupled release of membrane-derived cholesterol and phospholipids toward HDL particles, facilitating lipid redistribution and efflux [21]. Through its transporter function, ABCA1 drives the efflux of cholesterol originating from the breakdown of lipoproteins and its subsequent accumulation in the plasma membrane. The clearance of endosomal cholesterol relies on ABCA1-dependent retro-endocytosis of apo A1. Complementing this mechanism, ABCG1 provides an additional pathway that enhances cholesterol efflux and maintains lipid equilibrium [40]. ABCG1, a member of the ABC transporter family, functions in cholesterol export. Structurally, it possesses one NBD and TMD, characteristics that define it as a half transporter [46]. ABCG1 resides on the endoplasmic reticulum (ER) and participates in redistributing intracellular sterols, transferring cholesterol and oxysterols from the ER to the plasma membrane. At the cell surface, it channels cholesterol toward HDL and other lipid acceptors. The principal signaling pathways influenced by cholesterol dynamics are summarized in Table 1.

Mechanisms governing cholesterol efflux play a pivotal role in sustaining cancer cells’ viability and proliferation by maintaining intracellular cholesterol balance [54]. Multiple regulatory proteins, including ABCG1 [55], SR-B1 [56], LCAT, and apolipoprotein E (APOE), participate in this process, ultimately facilitating the organization of lipid raft microdomains essential for signal transduction.

**Figure 3:**
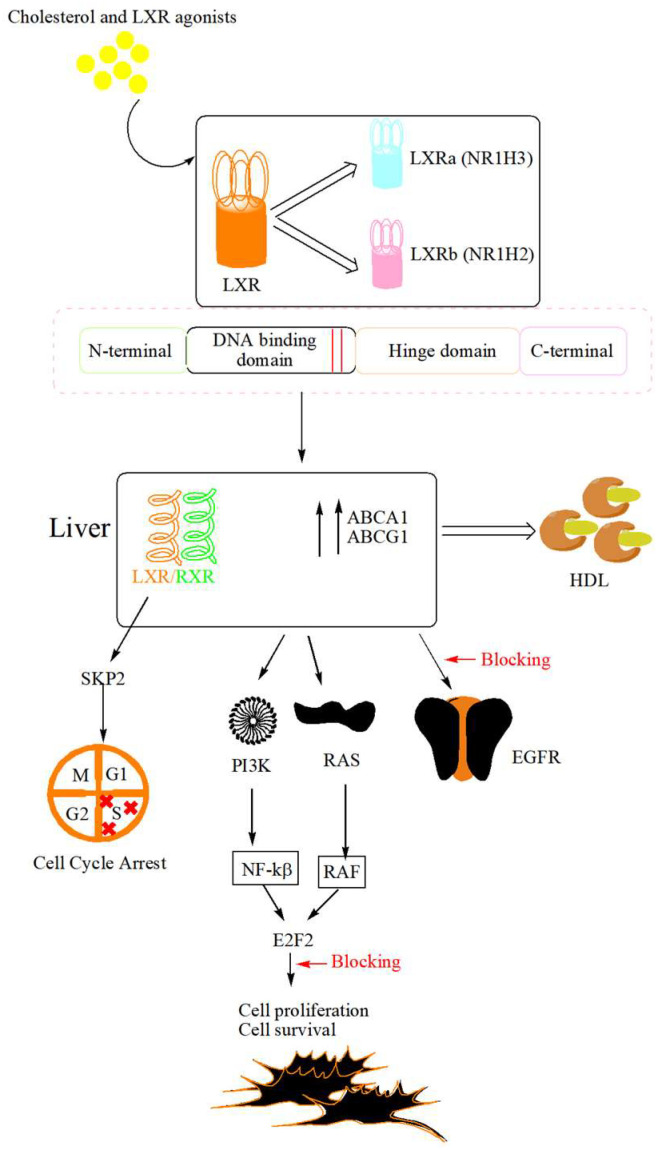
LXR in cholesterol homeostasis and activation of signaling pathways through activation. LXR is composed of two isoforms, LXRα and LXRβ, which regulate cholesterol and lipid homeostasis. Once the ligand gets attached to LXR, corepressor gets detached and activates the genes involved in cholesterol efflux, such as ABCA1 and ABCG1, and transports the cholesterol to HDL. Activation of LXR promotes cholesterol efflux and disrupts cholesterol-dependent oncogenic signaling, leading to cell cycle arrest, suppression of cancer cell proliferation, and reduced cell survival. Abbreviations: ABCA1: ATP-binding cassette subfamily A member 1; ABCG1, ATP-binding cassette subfamily G member 1; DNA, deoxyribonucleic acid; E2F2, E2F transcription factor 2; EGFR, epidermal growth factor receptor; HDL, high-density lipoprotein; LXR, liver X receptor; LXRα, liver X receptor α; LXRβ, liver X receptor β; NF-κB, nuclear factor kappa-light-chain-enhancer of activated B cells; NR1H2, nuclear receptor subfamily 1 group H member 2; NR1H3, nuclear receptor subfamily 1 group H member 3; PI3K, phosphoinositide 3-kinase; RAS, rat sarcoma virus; RAF, rapidly accelerated fibrosarcoma; RXR, retinoid X receptor; SKP2, S-phase kinase-associated protein 2.

Liver X receptors (LXRs), functioning as ligand-activated transcription factors within the nuclear receptor superfamily, are expressed as two isoforms—LXRα (nuclear receptor subfamily 1 group H member; NR1H3) and LXRβ (nuclear receptor subfamily 1 group H member 2; NR1H2) [57]. The principal role of LXRs is to maintain the cellular and systemic balance of cholesterol and lipid metabolism [58]. LXRα is mostly expressed in the liver, kidneys, and small intestine, with around 77–80% compared to the rest of the places like the adrenal, brain, heart, spleen, and testes [59,60]. The architecture of LXRs includes four distinct domains: the N-terminal activated function-1 (AF-1), a central DNA-binding domain, a hydrophobic ligand-binding pocket, and a C-terminal activation domain responsible for transcriptional regulation [61]. The LXR forms a heterodimer with the retinoid X receptor (RXR) that interacts with liver X-responsive elements (LXRE) within target gene promoters to modulate transcription in a ligand-dependent manner. In the unliganded state, the LXR/RXR complex associates with corepressor proteins, thereby suppressing gene expression. Upon binding of oxysterol ligands, conformational rearrangements within the receptor lead to the dissociation of corepressors and the recruitment of coactivator complexes. This transition initiates the transcription of downstream genes, including ABCA1 and ABCG1, which mediate cholesterol efflux to HDL [62,63]. The structural organization of LXR and its functional role in cancer are depicted in Figure 3.

The contributory role of LXRs in tumor progression is related to various types of activation and inhibition processes. Stimulation of LXR signaling results in a blockade of cell-cycle advancement from G1 to S phase, primarily through the reduced expression of S phase kinase-associated protein-2 (SKP2) [64,65]. The role of E2F transcription factor 2 (E2F2) has also been observed in estrogen receptor (ER)-positive breast cancer cell lines [66,67], showing constant downregulation of E2F2 after exposure to LXR agonists via inhibition of proliferation. LXR-mediated inhibition of cholesterol synthesis in prostate cancer systems, including both xenografted nude mice and in vitro cultures, affects the Ak strain transforming (Akt/protein kinase B), resulting in downstream dysregulation of signaling networks essential for cellular viability [47]. LXR activation in pancreatic ductal adenocarcinoma models interferes with growth factor signaling, prominently attenuating epidermal growth factor receptor (EGFR)-dependent pathways [52]. Furthermore, the transcriptional activation of melanoma cell secretion of APOE by LXR leads to inhibition of angiogenesis [53].

## 4. Correlation Between Cholesterol Metabolism and Cancer Progression

### 4.1. SREBP as a Potential Target

**Figure 4:**
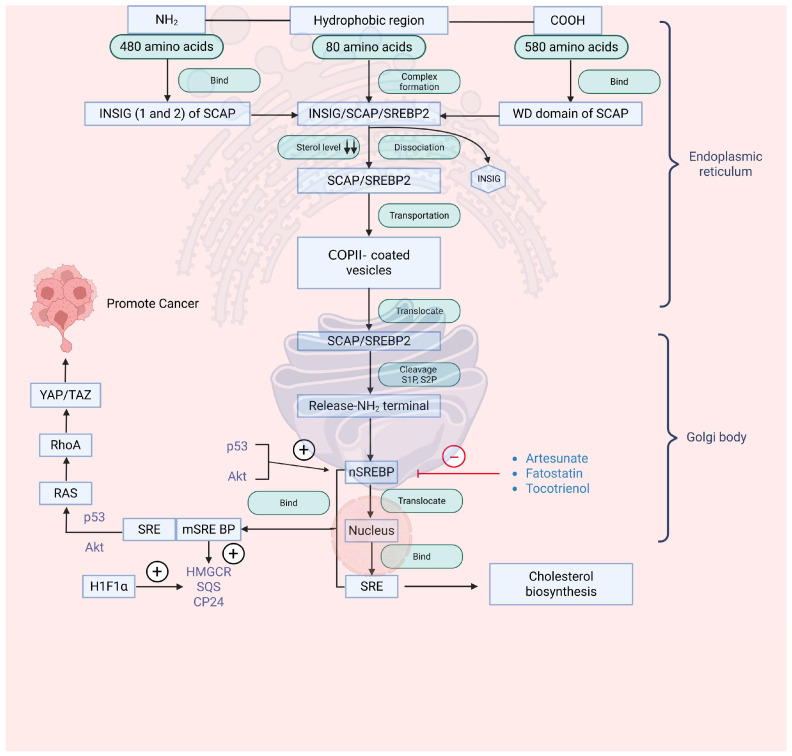
Regulation of SREBP2 in cancer and cholesterol. When level of sterol decreases, formation of SCAP/SREBP complex takes place by dissociating INSIG from INSIG/SCAP/SREBP2 complex. After this, COPII helps in transportation of complexes from ER to Golgi body. In Golgi apparatus, NH2 domain is released, and rest of the SREBP2 enters the nucleus, where it binds to SRE for cholesterol biosynthesis. Another way is that it binds with nSREBP, leading to cancer growth through RAS, RhoA, YAP/TAZ. Abbreviations: Akt, Ak strain transforming; COPII, coat protein complex II; CP24, 24-dehydrocholesterol reductase; HIF-1α, hypoxia-inducible factor-1 α; HMGCR, 3-hydroxy-3-methylglutaryl-CoA reductase; INSIG, insulin-induced gene protein; mSREBP, membrane-bound sterol regulatory element-binding protein; nSREBP, nuclear sterol regulatory element-binding protein; p53, tumor protein 53; RAS, rat sarcoma virus; RhoA, Ras homolog family member A; S1P, site-1 protease; S2P, site-2 protease; SCAP, SREBP cleavage-activating protein; SQS, squalene synthase; SRE, sterol regulatory element; SREBP, sterol regulatory element-binding protein; SREBP2, sterol regulatory element-binding protein 2; TAZ, transcriptional co-activator with PDZ-binding motif; WD, WD-repeat domain; YAP, Yes-associated protein.

SREBP-2 is a transcription factor required for maintaining cellular cholesterol balance [68]. It belongs to the SREBP family, which encompasses various isoforms, such as SREBP-1a and SREBP-1c [69,70], and acts as a major regulator of cholesterol metabolism in cellular homeostasis [1,71]. SREBP-2 consists of 1141 amino acids, an NH2 domain, a hydrophobic region in the middle, and a carboxyl group (COOH) at the terminal point [72]. The amine (NH2) domain at the terminal position contains DNA binding site known as the Bhlh-Zip motif. By binding to SP1 and NF-Y transcription factors, this regulatory element/motif contributes to the modulation of target gene expression. The middle region, comprising 80 amino acids, consists of a hydrophilic loop extending to the ER. Translocation and localization processes are carried out by the COOH domain. This COOH domain binds to WD40 (tryptophan (W) and aspartic acid (D)) of SREBP cleavage-activating protein (SCAP). In the ER, insulin-induced gene (INSIG), SCAP, and SREBP2 form a complex. Under low sterol levels, INSIG dissociates, allowing the SCAP/SREBP2 complex to be transported to COPII-coated vesicles from the ER to the Golgi apparatus. In the Golgi, site-1 and site-2 proteases (S1P and S2P) cleave SREBP2, releasing the active NH2-terminal fragment (nSREBP2). Following its activation, the processed fragment translocates to the nucleus and engages sterol regulatory elements (SREs) to enhance cholesterol biosynthetic activity. Concurrently, oncogenic networks, including RAS, RhoA, and YAP/TAZ signaling, facilitate malignant progression, as depicted in Figure 4.

SREBP2 expression is frequently altered in diverse tumors, notably in cancers of the breast, prostate, pancreas, and liver. The promotion of prostate cancer metastasis is associated with an aberrant lipogenic program dependent on SREBP, mainly when promyelocytic leukemia and the phosphatase and tensin homolog gene are simultaneously inactivated. This interplay helps in elucidating mechanisms for prostate cancer progression, possessing insights into potential targets for managing this cancer [73]. The significant correlation between elevated SREBP2 expression and unfavorable clinical outcomes in prostate cancer highlights the potential significance of SREBP2 as a prognostic indicator. Accumulating research indicates that heightened SREBP2 expression observed in advanced prostate cancer implicates this transcription factor in the regulation of tumor growth and progression [74]. The involvement of SREBP in prostate cancer progression is largely attributed to their ability to activate transcriptional programs governed by c-Myc and Akt [74].

Despite the clinical success of lapatinib and trastuzumab as HER2-targeted treatments, resistance, whether intrinsic or acquired, emerges in a significant subset of HER2-positive breast cancers [75,76,77,78]. Efforts to overcome this resistance have centered on modulating the mevalonate pathway, aiming to inhibit mechanistic target of rapamycin complex 1 (mTORC1) and SREBP2-mediated stimulation of the YAP/TAZ (Yes-associated protein/WW domain-containing transcription regulator 1) axis [79]. Zhang and co-workers [48] elucidated a novel mechanism in which the interaction between SREBP2 and transcription factor CP2 (TFCP2) activates HMGCR expression by inhibiting TFCP2-mediated senescence in prostate cancer cells [48]. ABCA9, another cholesterol transporter, has been found to inhibit the translocation of ER to the nucleus via Phosphatidylinositol 3-kinase (PI3K)-Akt-Forkhead Box O1 (FOXO1) pathway in breast cancer cells. ABCA9 operates as a cholesterol transporter in the ER, where it restrains sterol biosynthesis by attenuating SREBP2 activity. Reestablishing ABCA9 function markedly impedes the growth of breast cancer cells [49]. Pharmacological or genetic inhibition of eukaryotic elongation factor 2 kinase (EEF2K) hampers cholesterol biosynthesis by attenuating SREBP2-mediated signaling, and effect that can be mitigated by external cholesterol supply. This highlights the potential importance of targeting EEF2K to modulate cholesterol metabolism in cancer. Selective inhibition of EEF2K reduces tumor formation by downregulating SREBP2-mediated transcription of cholesterol-biosynthetic genes [50]. Attachment of X-box binding protein 1 (XBP1) to SREBP2 enhances 3-hydroxy-3-methylglutaryl-CoA reductase (HMGCR) activity, fostering cancer progression. Hence, simultaneous inhibition of SREBP2 and its co-regulatory elements may provide a valuable therapeutic avenue against cholesterol-driven tumorigenesis [80].

**Figure 5:**
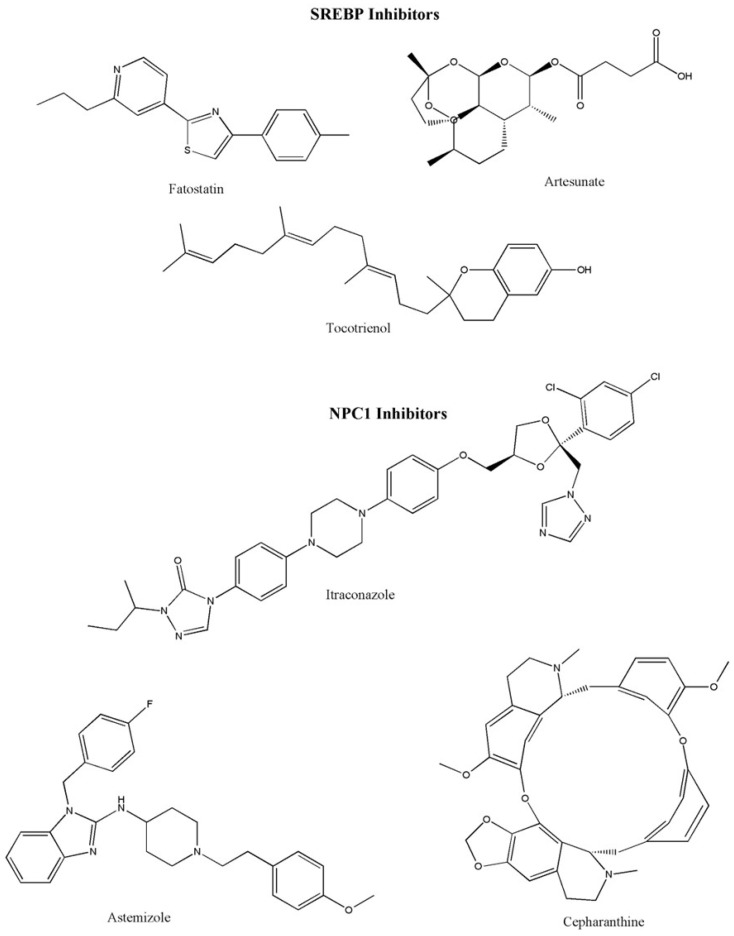
SREBP and NPC1 inhibitors. Abbreviations: NPC1, Niemann–Pick type C-1; SREBP, sterol regulatory element-binding protein 2.

Similarly, the drug artesunate (used for the treatment of malaria) is shown to inhibit the localization of SREBP2 by downregulating isopentenyl pyrophosphate (IPP) and glutathione peroxidase 4 (GPX4) in myeloma cells [81]. γ-Tocotrienol is another drug that decreases the level of triacylglycerol and cholesterol in prostate cells and rat hepatocytes [82,83]. These results emphasize that focusing on SREBP-2 and the mevalonate pathways can become a promising approach in the field of cancer therapy. Various SREBP1 inhibitors are displayed in Figure 5.

### 4.2. Niemann–Pick Type C1 as a Potential Target

The lysosomal membrane protein NPC1 governs the efflux of cholesterol and sphingolipids from the lysosomal lumen to the cytoplasm [84,85]. Through this trafficking function, NPC1 is essential for maintaining cholesterol equilibrium and preventing intracellular lipid overload. The NPC1 gene mutation causes various genetic disorders and also leads to the formation of cancer [86].

When cholesterol binds to late endosomes/lysosomes (LE/Lys), NPC2 transfers cholesterol to NPC1. Engagement of cholesterol with the sterol-sensing domain of NPC1 initiates mTOR signaling [87]. Activated SREBP, in turn, upregulates ABCA1 to enhance cholesterol import through LDL [88]. The exported lysosomal cholesterol is subsequently trafficked to the Golgi apparatus for distribution.

In response to reduced cellular cholesterol, steroidogenic acute regulatory-related lipid transfer domain-3 (STARD3) acts as a molecular shuttle, directing cholesterol trafficking from the ER to the lysosomal–endosome system [89], leading to activation of Ras-related protein Rab-7 (RAB7)/guanosine triphosphatase (GTPase) by forming the RAB7/TBC1D15 complex [90,91], which ultimately promotes metastasis.

**Figure 6:**
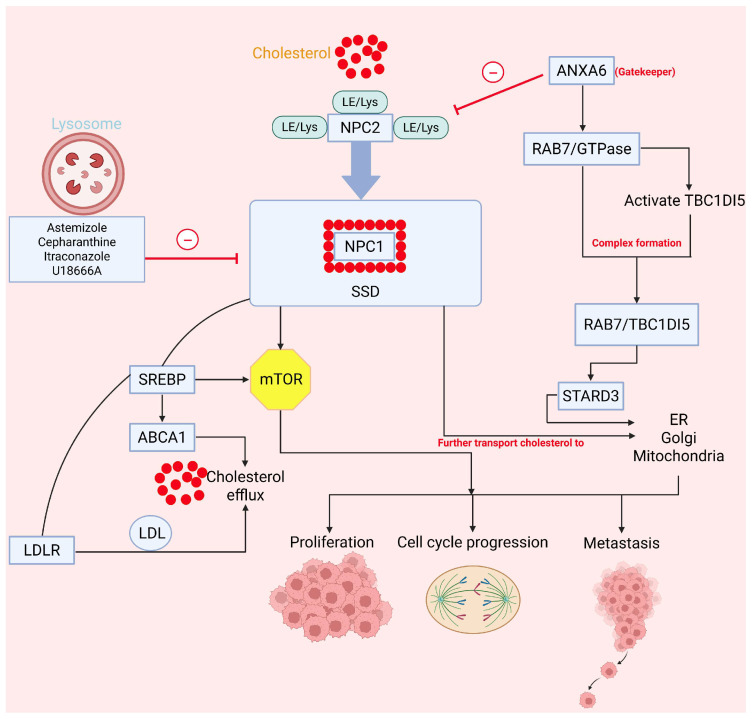
Signaling pathway of NPC1. NPC1/2 are both responsible for transfer of cholesterol from LE/lysosome area. When cholesterol binds with NPC2, it gets converted into NPC1 with SSD domain. It activates the mTOR and SREBP. SREBP activates the ABCA1 and LDLR, causing cholesterol efflux and its accumulation. All these factors lead to cell proliferation, progression, and metastasis. Abbreviations: ABCA1, ATP-binding cassette subfamily A member 1; ANXA6, annexin A6; ER, endoplasmic reticulum; GTPase, guanosine triphosphatase; LDL, low-density lipoprotein; LDLR, low-density lipoprotein receptor; LE/Lys, late endosome/lysosome; mTOR, mechanistic target of rapamycin; NPC1, Niemann–Pick type C1 protein; NPC2, Niemann–Pick type C2 protein; RAB7, Ras-related in brain protein 7; SREBP, sterol regulatory element-binding protein; SSD, sterol-sensing domain; STARD3, StAR-related lipid-transfer domain-containing protein 3; TBC1D5, TBC1 domain family member 5.

The researchers identified myeloid zinc Finger 1 (MZF1) as a controller of NPC1 expression induced by p95ErbB2, suggesting that ErbB2 signaling activation and NPC1 are essential in the cholesterol metabolism of cancer cells [92]. The link between MZF1 and NPC1 was reinforced through quantitative imaging-based cytometry (QIBC) analysis, revealing a notable decrease in cytosolic NPC1 levels in MZF1-knockdown cells or cells depleted of MZF1. According to Gene Ontology Biological Process (GOBP) enrichment, p95ErbB2-expressing cells display elevated expression of genes involved in cholesterol efflux, suggesting altered cellular cholesterol handling and homeostasis, potentially linked to changes in cholesterol transport [92]. The consequential hyper-activation of mTORC1 upon cholesterol accumulation due to NPC1 inhibition suggests a complex regulatory relationship that warrants further investigation into the underlying molecular mechanisms and potential implications for cellular functions [93]. In the presence of NPC1/2, lysosome-associated membrane protein (LAMP) serves as a crucial membrane component in lysosomes, which causes exocytosis, chaperone-mediated autophagy, or mTORC1 signaling [94]. Long and colleagues elucidated the binding site and mechanism by which itraconazole inhibits NPC1’s cholesterol-transport function [51]. They found, using cryo-electron microscopy (cryo-EM), that the 3D structure of NPC1 bound to itraconazole revealed the drug occupying the luminal cavity adjacent to the sterol-sensing domain (SSD), thereby blocking cholesterol transport; this was further confirmed by functional assays. The inhibitory mechanism closely resembles that of NPC1 blockade observed in the Hedgehog signaling pathway [51]. U18666A (Figure 5) is another small-molecule NPC1 inhibitor that suppresses the proliferation of triple-negative breast cancer cells and shows a synergistic effect with paclitaxel [86]. Cepharanthine and astemizole are additional NPC1 inhibitors that further disrupt cholesterol trafficking [95]. Figure 6 illustrates the crosstalk between cholesterol metabolism and cancer driven by NPC1 activity.

### 4.3. Sterol-Acyltransferase 1 in Cholesterol Metabolism and Cancer

**Figure 7:**
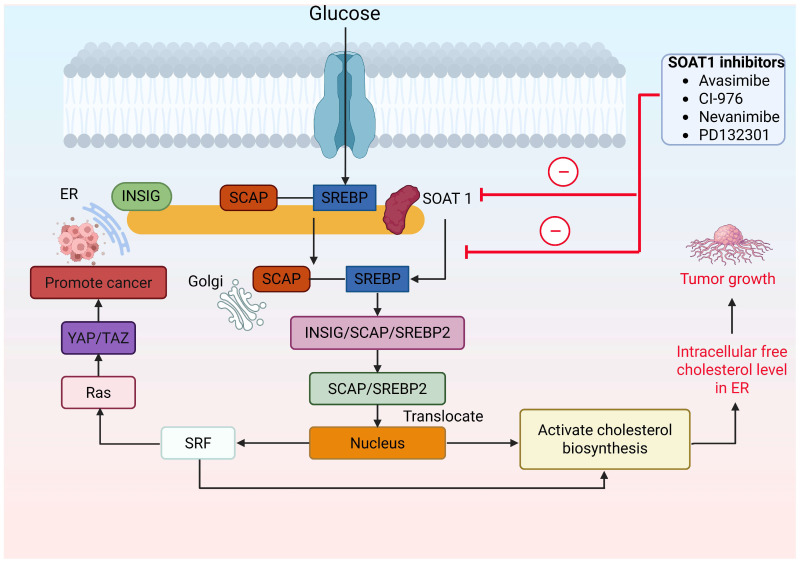
Regulatory pathway of SOAT1 with other components. Various factors activate the SCA/SREBP encapsulated in SOAT1 and INSIG, which translocate to Golgi. There, INSIG dissociates, and the rest of the complex translocates to the nucleus, where it activates the cholesterol biosynthesis. Along with this, it also activates SRF and RAS, which promote cancer. Abbreviations: ER, endoplasmic reticulum; INSIG, insulin-induced gene protein; Ras, rat sarcoma virus; SCAP, SREBP cleavage-activating protein; SOAT1, sterol O-acyltransferase 1; SRF, serum response factor; SREBP, sterol regulatory element-binding protein; SREBP2, sterol regulatory element-binding protein 2; TAZ, transcriptional co-activator with PDZ-binding motif; YAP, Yes-associated protein.

**Figure 8:**
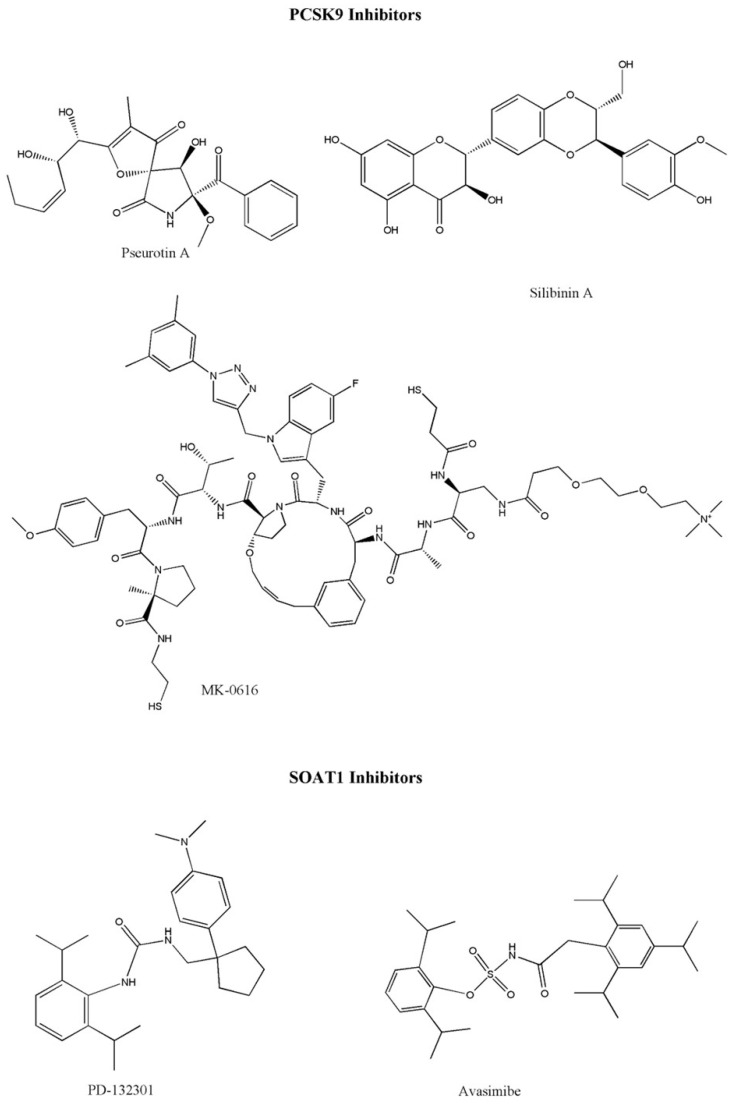
Various SOAT1 and PSCK9 inhibitors. Abbreviations: PCSK9, proprotein convertase subtilisin/kexin type 9; SOAT1, sterol-acyltransferase 1.

The enzyme SOAT1 plays a pivotal role in regulating cholesterol metabolism [96] through its function in cholesterol esterification, a fundamental step for the proper storage and intracellular distribution of cholesterol [97]. It is located in the ER and in tissues such as the liver and adipose. Alterations in SOAT1 induce a negative impact on cholesterol levels and the initiation of many physiological processes, such as gene mutation [98]. Free cholesterol in the ER controls SREBP2. When ER cholesterol is high (enhanced when SOAT1 is inhibited), INSIG binds the SCAP-SREBP2 complex to form an ER-retained INSIG-SCAP-SREBP2 complex, preventing SCAP from escorting SREBP2 to the Golgi and thus blocking SREBP activation. When the concentration of ER cholesterol falls, INSIG dissociates and SCAP ferries SREBP2 to the Golgi for proteolytic activation (Figure 7) [99]. Additionally, it activates the SRF, RAS, and YD/TAZ proteins, which promote cancer progression. There are several SOAT inhibitors that decrease the cholesterol accumulation. PD-132301 is one of the SOAT1 inhibitors that reduces the accumulation of cholesterol ester with a half-maximal inhibitory concentration (IC50) of 37 nM. In vivo studies showed that the introduction of PPPA derivatives to Apoe^−^/^−^ mice resulted in a significant reduction of total plasma cholesterol concentration of 57.9 ± 9.3% compared to the control group [100]. Nevanimibe hydrochloride is another SOAT1 inhibitor, which is given orally (1.6–158.5 mg/kg/day) with limited efficacy at a specific target [101]. Figure 8 shows the chemical structure of SOAT1 inhibitors. SOAT1 plays a crucial role in the mevalonate pathway, exerting negative feedback on unesterified cholesterol through p53 mutations in pancreatic cancer [96]. Xu and colleagues [102] reported that cholesterol activates the Wnt/PCP-YAP signaling pathway via SOAT1 in colon cancer. They also observed that cholesterol depletion lowers HMGCR levels, which in turn suppresses YAP activity, indicating YAP is dependent on cellular cholesterol levels.

### 4.4. PCSK9 as a Potential Target

**Figure 9:**
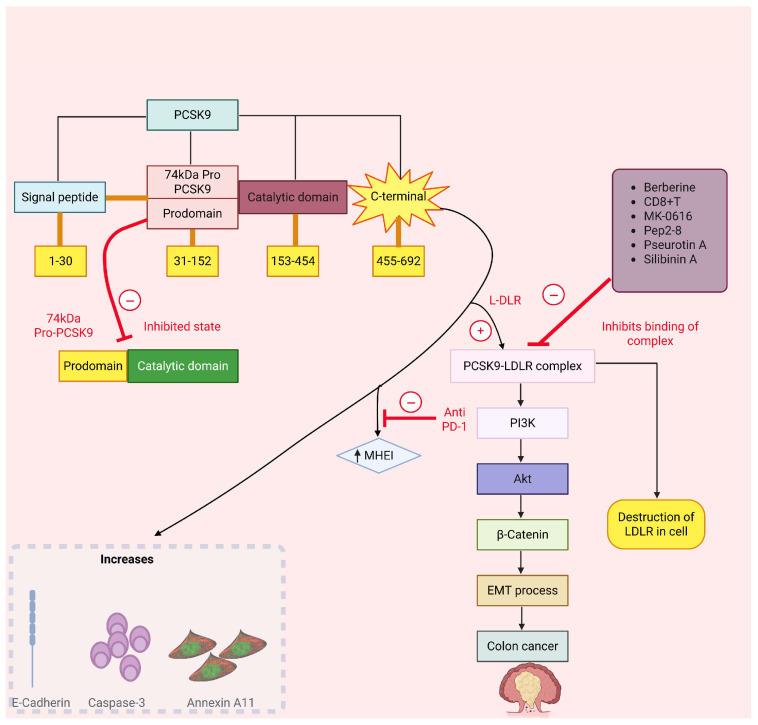
Signaling pathway of PCSK9 in cancer. PCSK9/LDLR complex activates the PI3K/Akt and other signaling cascades, which leads to initiation of EMT process and causes colon cancer. It also activates the SQLE, caspase-3, and E-cadherin, which are responsible for many types of cancer. Abbreviations: Akt, Ak strain transforming; CD8 + T, CD8-positive T lymphocyte; E-cadherin, epithelial cadherin; EMT, epithelial–mesenchymal transition; LDLR, low-density lipoprotein receptor; PCSK9: proprotein convertase subtilisin/kexin type 9; PD-1, programmed cell death protein 1; PI3K, phosphoinositide 3-kinase; SQLE, squalene epoxidase.

The enzyme PCSK9 contributes to hypercholesterolemia by modulating the activity of key lipid-regulatory genes, including LDLR and ApoB [103]. The biosynthesis of PCSK9 yields a 74 kDa proenzyme comprising a signal peptide (residues 1–30), an N-terminal prodomain (31–152), a catalytic core (153–454), and a C-terminal cysteine-rich region (455–692). In the ER, PCSK9 undergoes autocatalytic cleavage; the cleaved prodomain remains tightly bound as an intramolecular chaperone/inhibitor, and the secreted protein is catalytically inactive. The extracellular form of PCSK9 serves as a ligand for the LDL receptor (LDLR) by binding its EGF-A domain through the catalytic site. The C-terminal region directs the PCSK9-LDLR complex to endolysosomal compartments for degradation, diminishing surface levels of LDLR and VLDLR. Downstream, activation of the PCSK9-LDLR signaling cascade promotes PI3K-Akt-β-catenin pathway activity, induces epithelial–mesenchymal transition (EMT), and accelerates colon tumor progression, accompanied by alterations in caspase-3, Annexin A11, and E-cadherin expression. Small molecules and peptides (e.g., pseurotin A, silibinin A, MK-0616, Pep2–8, and berberine) can disrupt PCSK9-LDLR binding and prevent receptor destruction (Figure 9) [104,105,106]. Preclinical investigations revealed that phytochemical eugenol inhibits LDL by blocking PCSK9 through LOX1 inhibition [107,108]. Pseurotin A is one of the inhibitors that bind with PCSK9 in the interface pocket necessary for interaction with LDLR [108]. In vitro, pseurotin A reduces PCSK9 and boosts LDLR in hormone-dependent breast cancer cells, suggesting its potential as a dual inhibitor for hormone-dependent breast malignancies [107]. Figure 8 shows the chemical structure of various PCSK9 inhibitors. Currently, evolocumab and alirocumab are two PCSK9 inhibitor antibodies used to decrease lipid levels. These inhibitors exhibit adequate bioavailability (85% and 72%, respectively). Wong and co-workers [109] identified PCSK9 as a crucial determinant of cholesterol homeostasis and colorectal tumor progression under adenomatous polyposis coli (APC) and Kirsten rat sarcoma viral oncogene homolog (KRAS) mutations. In such tumors, PCSK9 downregulates cholesterol import while upregulating endogenous synthesis and elevating geranylgeranyl pyrophosphate (GGPP) levels, collectively enhancing KRAS/MEK/ERK signaling cascades. ERK1 and ERK2 were the most upregulated genes among the top 24 kinases. Various findings revealed that KRAS also activates p-MEK/p-ERK. MK-0616 is an orally available PCSK9 inhibitor that enhances the degradation of LDLR by binding with Toll-like receptors and other proteins [110,111]. It exerts its action by binding with the LDL-receptor and could act as a novel target in cancer. In a clinical trial with 380 treated participants, MK-0616 (6–30 mg) produced significant week-8 LDL-c reductions compared to placebo (−41.2%, −55.7%, −59.1%, −60.9%; all *p* < 0.001) with adverse event rates comparable to placebo (39.5–43.4% vs. 44.0%) [112].

## 5. Cholesterol Metabolism and Cancer Immunotherapy

Cholesterol metabolism has recently emerged as a critical regulator of antitumor immune responses, particularly in T cell-based immunotherapies. Emerging evidence indicates that cholesterol metabolism plays a crucial role in shaping antitumor immune responses and immunotherapy efficacy [113]. Beyond tumor-intrinsic effects, altered cholesterol homeostasis profoundly influences immune cell function within the tumor microenvironment. Recent studies have shown that excessive cholesterol accumulation in T cells can drive cellular exhaustion and impair cytotoxic activity, whereas controlled modulation of cholesterol flux enhances immune responsiveness. In this context, Su et al. [114] demonstrated that inhibition of cholesterol esterification through acetyl-CoA acetyltransferase-1 (ACAT1) knockdown markedly potentiates CD19-directed CAR-T cell therapy. ACAT1 inhibition increased free cholesterol levels in the plasma membrane, promoting T cell receptor clustering, improving immunological synapse formation, and enhancing downstream signaling. As a result, metabolically reprogrammed CAR-T cells exhibited increased proliferation, cytokine secretion (IFN-γ), degranulation, and superior tumor cell killing in lymphoma models [114].

Additional evidence further suggests that cholesterol metabolism interacts with immune checkpoint regulation, antigen presentation, and macrophage polarization. Dysregulated cholesterol synthesis and efflux pathways within the tumor microenvironment can suppress effector T cell activity while promoting immunosuppressive phenotypes such as exhausted T cells and tumor-associated macrophages. Targeting key nodes of cholesterol metabolism, such as ACAT1, SREBP signaling, or cholesterol transporters, has therefore emerged as a promising approach to strengthen antitumor immunity and enhance the therapeutic efficacy of immune checkpoint inhibitors and adoptive cell therapies. Collectively, these findings underscore cholesterol metabolic reprogramming as a novel and actionable axis for improving cancer immunotherapy outcomes [115,116,117,118].

## 6. Conclusions

**Figure 10:**
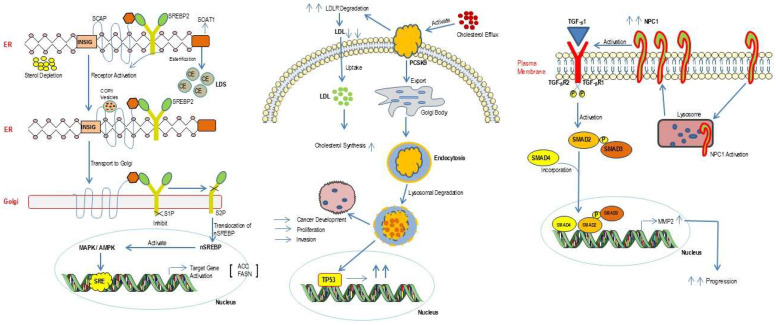
Integrated schematic demonstrating the role of cholesterol metabolic regulators (SREBP2, SOAT1, NPC1, and PCSK9) in tumor progression and therapy resistance. Cholesterol depletion activates SREBP2 via ER–Golgi processing, inducing genes involved in cholesterol synthesis, uptake, and esterification, including LDLR and SOAT1. Enhanced LDL uptake and SOAT1-mediated cholesterol storage support tumor cell proliferation and survival. PCSK9 regulates LDLR turnover and cholesterol flux, contributing to metabolic adaptation and therapy resistance. NPC1-dependent lysosomal cholesterol trafficking activates TGF-β/SMAD signaling, promoting invasion and tumor progression. Abbreviations: ACC, acety CoA carboxylase; AMPK, AMP-activated protein kinase; CE, cholesteryl ester(s); COPII, coat protein complex II; ER, endoplasmic reticulum; FASN, fatty acid synthase; INSIG, insulin-induced gene; LDL, low-density lipoprotein; LDLR, low-density lipoprotein receptor; LDS, lipid droplets; MAPK, mitogen-activated protein kinase; MMP2, matrix metalloproteinase 2; nSREBP, nuclear sterol regulatory-element binding protein; NPC1, Niemann–Pick disease type C1 protein; PCSK9, proprotein convertase subtilisin/kexin type 9; S1P, site-1 protease; S2P, site-2 protease; SCAP, SREBP cleavage-activating protein; SMAD2, SMAD family member 2; SMAD3, SMAD family member 3; SMAD4, SMAD family member 4; SOAT1, sterol-*O*-acyltransferase 1 (also known as ACAT1); SRE, sterol regulatory element; SREBP2, sterol regulatory element-binding protein 2; TGF-β1, transforming growth factor β1; TGF-βR1, transforming growth factor β receptor 1; TGF-βR2, transforming growth factor β receptor 2; and TP53, tumor protein p53.

Collectively, the interplay between cholesterol metabolism and cancer progression encompasses several key molecular pathways, including SREBP, NPC1, PCSK9, and SOAT1 (Figure 10). SREBP2 orchestrates lipid biosynthetic processes that foster tumor growth; SOAT1 mediates cholesterol esterification within tumor cells; NPC1 regulates intracellular cholesterol transport; and PCSK9 promotes LDL receptor degradation, resulting in elevated LDL-cholesterol levels. Perturbation of these regulatory axes contributes significantly to oncogenic advancement. Elucidating the mechanistic roles of these proteins not only enhances our understanding of tumor metabolism but also identifies cholesterol regulatory axes as compelling therapeutic targets. Accordingly, Table 2 briefly summarizes the therapeutic strategies for targeting cholesterol metabolism.

Accumulating clinical and experimental evidence underscores the pivotal role of altered cholesterol metabolism in oncogenesis. Deciphering the molecular interrelationships among cholesterol-regulating proteins offers a strong foundation for developing targeted metabolic interventions. In this context, drug repurposing represents a particularly attractive strategy, enabling the identification of anticancer applications for clinically approved lipid-lowering agents. Nevertheless, further in-depth studies are required to develop novel inhibitors, define new mechanisms of action, and clarify the role of cholesterol efflux-associated proteins in cancer initiation, progression, and therapeutic resistance.

### Future Perspectives

Future progress in this field will depend on addressing several critical knowledge gaps. These include elucidating the context-dependent functions of cholesterol-regulating proteins across distinct cancer subtypes and tumor microenvironments; developing highly selective modulators of SREBP2, SOAT1, NPC1, and PCSK9 with favorable safety profiles; and exploring comprehensive treatment strategies that combine cholesterol-targeting approaches with immunotherapy or iron death inducers, in order to overcome the resistance mechanisms. In parallel, systematic drug repurposing methods and patient stratification strategies based on biomarkers are crucial for advancing the application of precision oncology. Ultimately, large-scale and well-designed clinical trials are needed to translate the mechanistic insights into effective, patient-specific therapies. Collectively, continuous interdisciplinary collaboration and innovation can establish cholesterol metabolism as a transformative treatment frontier in oncology, offering new opportunities for improving patient outcomes.

## Figures and Tables

**Table 1:** Cholesterol-mediated signaling pathways in cancer.

Pathway	Cholesterol Role	Cancer Impact	Therapeutic Potential	Reference
Lipid Rafts	Organize membrane receptors	Enhances EGFR, HER2, and TCR signaling	Disruption impairs growth signaling	[47,48]
PI3K/Akt/mTOR	Raft stability and feedback to SREBP2	Promotes survival and growth	Statins and SREBP2 inhibitors	[49,50]
Hh	Cholesterol binds SMO	Drives proliferation in Hh-dependent tumors	SMO inhibitors and cholesterol synthesis inhibitors	[51]
LXR	Modulates cholesterol efflux, immune genes	Suppresses or promotes tumors contextually	LXR agonists/antagonists	[52,53]

Abbreviations: Akt, Ak strain transforming; EGFR, epidermal growth factor receptor; HER2, human epidermal growth factor receptor type 2; Hh, Hedgehog signaling protein; LXR, liver X receptor; mTOR, mechanistic target of rapamycin; PI3K, phosphoinositide 3-kinase; SMO, smoothened receptor; SREBP2, sterol regulatory element-binding protein 2; TCR, T cell receptor.

**Table 2:** Therapeutic strategies targeting cholesterol metabolism.

Strategy	Target	Example Agents	Mechanism	Associated Disease	Development Stage	Reference
Synthesis inhibition	HMGCR	Statins (simvastatin)	Blocks mevalonate pathway	Pancreatic Cancer	Clinical (mixed results)	[48]
Synthesis inhibition	SQLE	NB-598 and terbinafine	Inhibits downstream cholesterol synthesis	Not Reported	Preclinical/repurposing	[119]
Uptake inhibition	LDLR	MK-0616	Reduces exogenous cholesterol acquisition	Hypercholesterolemia	Preclinical	[112]
Intracellular transport	NPC1	Itraconazole and U18666A	Disrupts lysosomal cholesterol export	Breast Cancer	Preclinical	[86]
Esterification inhibition	ACAT1	K-604	Blocks cholesterol storage	Glioblastoma	Preclinical	[120]
Efflux enhancement	LXRs	GW3965 and RGX-104	Induces ABCA1/ABCG1 and modulates immunity	Glioblastoma	Early clinical/preclinical	[53,121]
Nanocarrier delivery	Not reported	Lipid NPs and micelles	Exerts targeted delivery of cholesterol modulators	Breast Cancer	Experimental	[122]
Epigenetic/genetic targeting	SREBP2 and ACAT1	CRISPR and siRNA	Disrupts key regulators of cholesterol homeostasis	Melanoma	Experimental	[80,123]
Dietary/microbiome approaches	Systemic cholesterol	Statins with probiotics	Modulates systemic and tumor cholesterol	Colorectal Cancer	Emerging concept	[124]

Abbreviations: ABCA1, ATP-binding cassette transporter-1; LDLR, low-density lipoprotein receptor; NPC1, Niemann–Pick type C-1; NPs, nanoparticles; SREBP2, sterol regulatory element-binding protein 2.

## Data Availability

Data will be made available on request.

## Abbreviations

ABCA1	ATP-binding cassette subfamily A1
ABCG1	ATP-binding cassette subfamily G1
Acetyl-CoA	Acetyl coenzyme A
Akt	Ak strain transforming
ANXA6	Annexin A6
APC	Adenomatous polyposis coli
APOE	Apolipoprotein E
CD8 + T	CD8-positive T lymphocyte
COPII	Coat protein complex II
CP24	24-dehydrocholesterol reductase (DHCR24)
CRISPR	Clustered regularly interspaced short palindromic repeats
cryo-EM	Cryo-electron microscopy
DHCR24	(Standard for CP24) 24-dehydrocholesterol reductase
DMAPP	Dimethylallyl pyrophosphate
DNA	Deoxyribonucleic acid
E-cadherin	Epithelial cadherin
E2F2	E2F transcription factor 2
EEF2K	Eukaryotic elongation factor 2 kinase
EGFR	Epidermal growth factor receptor
EMT	Epithelial–mesenchymal transition
ER	Endoplasmic reticulum
ERK	Extracellular signal-regulated kinase
FOXO1	Forkhead Box O1
GGPP	Geranylgeranyl pyrophosphate
GOBP	Gene ontology biological process
GPX4	Glutathione peroxidase 4
GTPase	Guanosine triphosphatase
HDL	High-density lipoprotein
HER2	Human epidermal growth factor receptor type 2
Hh	Hedgehog signaling protein
HIF-1α	Hypoxia-inducible factor-1 α
HMG-CoA	3-hydroxy-3-methylglutaryl coenzyme A
HMGCR	3-hydroxy-3-methylglutaryl-CoA reductase
INSIG	Insulin-induced gene protein
IPP	Isopentyl pyrophosphate
KRAS	Kirsten rat sarcoma viral oncogene homolog
LAMP	Lysosome-associated membrane protein
LCAT	Lecithin–cholesterol acyltransferase
LDL	Low-density lipoprotein
LDLR	Low-density lipoprotein receptor
LE/Lys	Late endosome/lysosome
LOX1	Lectin-like oxidized low-density lipoprotein receptor-1
LXR	Liver X receptor
LXRE	Liver X-responsive elements
LXRα	Liver X receptor α (NR1H3)
LXRβ	Liver X receptor β (NR1H2)
MEK	Mitogen-activated protein kinase kinase
mSREBP	Membrane-bound sterol regulatory element-binding protein
mTOR	Mechanistic target of rapamycin
mTORC1	Mechanistic target of rapamycin complex 1
MZF1	Myeloid zinc Finger 1
NBD	Nucleotide-binding domains
NF-κB	Nuclear factor kappa-light-chain-enhancer of activated B cells
NPC1	Niemann–Pick type C1 protein
NPC2	Niemann–Pick type C2 protein
NPs	Nanoparticles
NR1H2	Nuclear receptor subfamily 1 group H member 2 (LXRβ)
NR1H3	Nuclear receptor subfamily 1 group H member 3 (LXRα)
nSREBP	Nuclear sterol regulatory element-binding protein
p53	Tumor protein 53
PCSK9	Proprotein convertase subtilisin/kexin type 9
PD-1	Programmed cell death protein 1
PI3K	Phosphoinositide 3-kinase
QIBC	Quantitative imaging-based cytometry
RAB7	Ras-related in brain protein 7
RAF	Rapidly accelerated fibrosarcoma
RAS	Rat sarcoma virus
RCT	Reverse cholesterol transport
RhoA	Ras homolog family member A
RXR	Retinoid X receptor
S1P	Site-1 protease
S2P	Site-2 protease
SCAP	SREBP cleavage-activating protein
siRNA	Small interfering RNA
SKP2	S-phase kinase-associated protein 2
SMO	Smoothened receptor
SOAT1	Sterol O-acyltransferase 1
SQLE	Squalene epoxidase
SQS	Squalene synthase
SR-B1	Scavenger receptor class B type 1
SRE	Sterol regulatory element
SREBP	Sterol regulatory element-binding protein
SREBP2	Sterol regulatory element-binding protein 2
SRF	Serum response factor
SSD	Sterol-sensing domain
STARD3	StAR-related lipid-transfer domain-containing protein 3
TAZ	Transcriptional co-activator with PDZ-binding motif
TBC1D5	TBC1 domain family member 5
TCR	T cell receptor
TFCP2	Transcription factor CP2
TMD	Transmembrane domains
VLDL	Very low-density lipoprotein
VLDLR	Very low-density lipoprotein receptor
WD	WD-repeat domain
XBP1	X-box binding protein 1
YAP	Yes-associated protein

## References

[1] Luo J., Yang H., Song B.L. (2020). Mechanisms and regulation of cholesterol homeostasis. Nat. Rev. Mol. Cell Biol..

[2] Martin-Perez M., Urdiroz-Urricelqui U., Bigas C., Benitah S.A. (2022). The role of lipids in cancer progression and metastasis. Cell Metab..

[3] Riscal R., Skuli N., Simon M.C. (2019). Even Cancer Cells Watch Their Cholesterol!. Mol. Cell.

[4] Ikonen E. (2008). Cellular cholesterol trafficking and compartmentalization. Nat. Rev. Mol. Cell Biol..

[5] Carson J.A.S., Lichtenstein A.H., Anderson C.A.M., Appel L.J., Kris-Etherton P.M., Meyer K.A., Petersen K., Polonsky T., Van Horn L. (2020). Dietary Cholesterol and Cardiovascular Risk: A Science Advisory From the American Heart Association. Circulation.

[6] Duran E.K., Aday A.W., Cook N.R., Buring J.E., Ridker P.M., Pradhan A.D. (2020). Triglyceride-Rich Lipoprotein Cholesterol, Small Dense LDL Cholesterol, and Incident Cardiovascular Disease. J. Am. Coll. Cardiol..

[7] Das P., Ingole N. (2023). Lipoproteins and Their Effects on the Cardiovascular System. Cureus.

[8] Groenen A.G., Halmos B., Tall A.R., Westerterp M. (2021). Cholesterol efflux pathways, inflammation, and atherosclerosis. Crit. Rev. Biochem. Mol. Biol..

[9] Chen F., Lu Y., Lin J., Kang R., Liu J. (2023). Cholesterol Metabolism in Cancer and Cell Death. Antioxid. Redox Signal..

[10] Varsano N., Capua-Shenkar J., Leiserowitz L., Addadi L. (2022). Crystalline cholesterol: The material and its assembly lines. Annu. Rev. Mater. Res..

[11] Jung S.M., Kang D., Guallar E., Yu J., Lee J.E., Kim S.W., Nam S.J., Cho J., Lee S.K. (2020). Impact of Serum Lipid on Breast Cancer Recurrence. J. Clin. Med..

[12] Duan Y., Gong K., Xu S., Zhang F., Meng X., Han J. (2022). Regulation of cholesterol homeostasis in health and diseases: From mechanisms to targeted therapeutics. Signal Transduct. Target. Ther..

[13] Fernández L.P., Gómez de Cedrón M., Ramírez de Molina A. (2020). Alterations of Lipid Metabolism in Cancer: Implications in Prognosis and Treatment. Front. Oncol..

[14] Marino N., German R., Rao X., Simpson E., Liu S., Wan J., Liu Y., Sandusky G., Jacobsen M., Stoval M. (2020). Upregulation of lipid metabolism genes in the breast prior to cancer diagnosis. NPJ Breast Cancer.

[15] Göbel A., Rauner M., Hofbauer L.C., Rachner T.D. (2020). Cholesterol and beyond—The role of the mevalonate pathway in cancer biology. Biochim. Biophys. Acta Rev. Cancer.

[16] Liu W., Chakraborty B., Safi R., Kazmin D., Chang C.-Y., McDonnell D.P. (2021). Dysregulated cholesterol homeostasis results in resistance to ferroptosis increasing tumorigenicity and metastasis in cancer. Nat. Commun..

[17] Vona R., Iessi E., Matarrese P. (2021). Role of Cholesterol and Lipid Rafts in Cancer Signaling: A Promising Therapeutic Opportunity?. Front. Cell Dev. Biol..

[18] Barbalata C.I., Tefas L.R., Achim M., Tomuta I., Porfire A.S. (2020). Statins in risk-reduction and treatment of cancer. World J. Clin. Oncol..

[19] Tian Y., Li J., Yang X. (2025). Statins as potential adjuvant therapy in lung cancer: A narrative review. J. Thorac. Dis..

[20] Guo X.J., Zhu B.B., Li J., Guo P., Niu Y.B., Shi J.L., Yokoyama W., Huang Q.S., Shao D.Y. (2025). Cholesterol metabolism in tumor immunity: Mechanisms and therapeutic opportunities for cancer. Biochem. Pharmacol..

[21] Sharma B., Agnihotri N. (2019). Role of cholesterol homeostasis and its efflux pathways in cancer progression. J. Steroid Biochem. Mol. Biol..

[22] Schade D.S., Shey L., Eaton R.P. (2020). Cholesterol Review: A Metabolically Important Molecule. Endocr. Pract..

[23] Cockcroft S. (2021). Mammalian lipids: Structure, synthesis and function. Essays Biochem..

[24] Skeyni A., Pradignac A., Matz R.L., Terrand J., Boucher P. (2024). Cholesterol trafficking, lysosomal function, and atherosclerosis. Am. J. Physiol. Cell Physiol..

[25] Meng Y., Heybrock S., Neculai D., Saftig P. (2020). Cholesterol Handling in Lysosomes and Beyond. Trends Cell Biol..

[26] McDonald S. (2022). Systematic Study of Cellular Cholesterol Homeostasis. Master’s Thesis.

[27] van der Wulp M.Y., Verkade H.J., Groen A.K. (2013). Regulation of cholesterol homeostasis. Mol. Cell. Endocrinol..

[28] Ho W.Y., Hartmann H., Ling S.C. (2022). Central nervous system cholesterol metabolism in health and disease. IUBMB life.

[29] Ridgway N., McLeod R. (2008). Biochemistry of Lipids, Lipoproteins and Membranes.

[30] Cui D., Yu X., Guan Q., Shen Y., Liao J., Liu Y., Su Z. (2025). Cholesterol metabolism: Molecular mechanisms, biological functions, diseases, and therapeutic targets. Mol. Biomed..

[31] Cerqueira N.M., Oliveira E.F., Gesto D.S., Santos-Martins D., Moreira C., Moorthy H.N., Ramos M.J., Fernandes P.A. (2016). Cholesterol Biosynthesis: A Mechanistic Overview. Biochemistry.

[32] Bhale A.S., Meilhac O., d’Hellencourt C.L., Vijayalakshmi M.A., Venkataraman K. (2024). Cholesterol transport and beyond: Illuminating the versatile functions of HDL apolipoproteins through structural insights and functional implications. Biofactors.

[33] Litvinov D.Y., Savushkin E.V., Garaeva E.A., Dergunov A.D. (2016). Cholesterol Efflux and Reverse Cholesterol Transport: Experimental Approaches. Curr. Med. Chem..

[34] Ouimet M., Barrett T.J., Fisher E.A. (2019). HDL and reverse cholesterol transport: Basic mechanisms and their roles in vascular health and disease. Circ. Res..

[35] Pownall H.J., Rosales C., Gillard B.K., Gotto A.M. (2021). High-density lipoproteins, reverse cholesterol transport and atherogenesis. Nat. Rev. Cardiol..

[36] Dergunov A.D., Baserova V.B. (2022). Different pathways of cellular cholesterol efflux. Cell Biochem. Biophys..

[37] Litvinov D.Y., Savushkin E.V., Dergunov A.D. (2018). Intracellular and plasma membrane events in cholesterol transport and homeostasis. J. Lipids.

[38] Brufau G., Groen A.K., Kuipers F. (2011). Reverse cholesterol transport revisited: Contribution of biliary versus intestinal cholesterol excretion. Arterioscler. Thromb. Vasc. Biol..

[39] Khattib A., Shmet M., Ashkar R., Hayek T., Khatib S. (2024). Novel bioactive lipids enhanced HDL-mediated cholesterol efflux from macrophages through the ABCA1 receptor pathway. Chem. Phys. Lipids.

[40] Tall A.R. (2008). Cholesterol efflux pathways and other potential mechanisms involved in the athero-protective effect of high density lipoproteins. J. Intern. Med..

[41] Holland I.B. (2019). Rise and rise of the ABC transporter families. Res. Microbiol..

[42] Chen L., Zhao Z.W., Zeng P.H., Zhou Y.J., Yin W.J. (2022). Molecular mechanisms for ABCA1-mediated cholesterol efflux. Cell Cycle.

[43] Liu Y., Tang C. (2012). Regulation of ABCA1 functions by signaling pathways. Biochim. Biophys. Acta (BBA) Mol. Cell Biol. Lipids.

[44] Maïga S.F., Kalopissis A.-D., Chabert M. (2014). Apolipoprotein A-II is a key regulatory factor of HDL metabolism as appears from studies with transgenic animals and clinical outcomes. Biochimie.

[45] Sánchez S.A., Tricerri M.A., Ossato G., Gratton E. (2010). Lipid packing determines protein-membrane interactions: Challenges for apolipoprotein A-I and high density lipoproteins. Biochim. Biophys. Acta.

[46] Xu D., Li Y., Yang F., Sun C., Pan J., Wang L., Chen Z., Fang S., Yao X., Hou W. (2022). Structure and transport mechanism of the human cholesterol transporter ABCG1. Cell Rep..

[47] Piccinin E., Arconzo M., Pasculli E., Tricase A.F., Cultrera S., Bertrand-Michel J., Loiseau N., Villani G., Guillou H., Moschetta A. (2024). Pivotal role of intestinal cholesterol and nuclear receptor LXR in metabolic liver steatohepatitis and hepatocarcinoma. Cell Biosci..

[48] Zhang D., Lu P., Zhu K., Wu H., Dai Y. (2021). TFCP2 Overcomes Senescence by Cooperating With SREBP2 to Activate Cholesterol Synthesis in Pancreatic Cancer. Front. Oncol..

[49] Hwang H.-J., Lee K.-H., Cho J.-Y. (2023). ABCA9, an ER cholesterol transporter, inhibits breast cancer cell proliferation via SREBP-2 signaling. Cancer Sci..

[50] Zhao J., Zhang X., Gao T., Wang S., Hou Y., Yuan P., Yang Y., Yang T., Xing J., Li J. (2020). SIK2 enhances synthesis of fatty acid and cholesterol in ovarian cancer cells and tumor growth through PI3K/Akt signaling pathway. Cell Death Dis..

[51] Long T., Qi X., Hassan A., Liang Q., De Brabander J.K., Li X. (2020). Structural basis for itraconazole-mediated NPC1 inhibition. Nat. Commun..

[52] Chen T., Xu J., Fu W. (2020). EGFR/FOXO3A/LXR-α Axis Promotes Prostate Cancer Proliferation and Metastasis and Dual-Targeting LXR-α/EGFR Shows Synthetic Lethality. Front. Oncol..

[53] Tavazoie M.F., Pollack I., Tanqueco R., Ostendorf B.N., Reis B.S., Gonsalves F.C., Kurth I., Andreu-Agullo C., Derbyshire M.L., Posada J. (2018). LXR/ApoE Activation Restricts Innate Immune Suppression in Cancer. Cell.

[54] Xiao M., Xu J., Wang W., Zhang B., Liu J., Li J., Xu H., Zhao Y., Yu X., Shi S. (2023). Functional significance of cholesterol metabolism in cancer: From threat to treatment. Exp. Mol. Med..

[55] Kennedy M.A., Barrera G.C., Nakamura K., Baldán Á., Tarr P., Fishbein M.C., Frank J., Francone O.L., Edwards P.A. (2005). ABCG1 has a critical role in mediating cholesterol efflux to HDL and preventing cellular lipid accumulation. Cell Metab..

[56] Shen W.-J., Azhar S., Kraemer F.B. (2018). SR-B1: A unique multifunctional receptor for cholesterol influx and efflux. Annu. Rev. Physiol..

[57] Calkin A.C., Tontonoz P. (2012). Transcriptional integration of metabolism by the nuclear sterol-activated receptors LXR and FXR. Nat. Rev. Mol. Cell Biol..

[58] Wang B., Tontonoz P. (2018). Liver X receptors in lipid signalling and membrane homeostasis. Nat. Rev. Endocrinol..

[59] Ducheix S., Montagner A., Theodorou V., Ferrier L., Guillou H. (2013). The liver X receptor: A master regulator of the gut–liver axis and a target for non alcoholic fatty liver disease. Biochem. Pharmacol..

[60] Song X., Wu W., Warner M., Gustafsson J.-Å. (2022). Liver X receptor regulation of glial cell functions in the CNS. Biomedicines.

[61] Jakobsson T., Treuter E., Gustafsson J.-Å., Steffensen K.R. (2012). Liver X receptor biology and pharmacology: New pathways, challenges and opportunities. Trends Pharmacol. Sci..

[62] Zelcer N., Hong C., Boyadjian R., Tontonoz P. (2009). LXR regulates cholesterol uptake through Idol-dependent ubiquitination of the LDL receptor. Science.

[63] Danielewski M., Rapak A., Kruszyńska A., Małodobra-Mazur M., Oleszkiewicz P., Dzimira S., Kucharska A.Z., Słupski W., Matuszewska A., Nowak B. (2024). Cornelian cherry (*Cornus mas* L.) fruit extract lowers SREBP-1c and C/EBPα in liver and alters various PPAR-α, PPAR-γ, LXR-α target genes in cholesterol-rich diet rabbit model. Int. J. Mol. Sci..

[64] Kim K.H., Lee G.Y., Kim J.I., Ham M., Won Lee J., Kim J.B. (2010). Inhibitory effect of LXR activation on cell proliferation and cell cycle progression through lipogenic activity. J. Lipid Res..

[65] Meng Z.X., Yin Y., Lv J.H., Sha M., Lin Y., Gao L., Zhu Y.X., Sun Y.J., Han X. (2012). Aberrant activation of liver X receptors impairs pancreatic beta cell function through upregulation of sterol regulatory element-binding protein 1c in mouse islets and rodent cell lines. Diabetologia.

[66] Nguyen-Vu T., Vedin L.-L., Liu K., Jonsson P., Lin J.Z., Candelaria N.R., Candelaria L.P., Addanki S., Williams C., Gustafsson J.-Å.J. (2013). Liver× receptor ligands disrupt breast cancer cell proliferation through an E2F-mediated mechanism. Breast Cancer Res..

[67] Wu Y., Yu D.D., Yan D.L., Hu Y., Chen D., Liu Y., Zhang H.D., Yu S.R., Cao H.X., Feng J.F. (2016). Liver X receptor as a drug target for the treatment of breast cancer. Anti Cancer Drugs.

[68] Griffiths B., Lewis C.A., Bensaad K., Ros S., Zhang Q., Ferber E.C., Konisti S., Peck B., Miess H., East P. (2013). Sterol regulatory element binding protein-dependent regulation of lipid synthesis supports cell survival and tumor growth. Cancer Metab..

[69] Shimano H., Sato R. (2017). SREBP-regulated lipid metabolism: Convergent physiology—Divergent pathophysiology. Nat. Rev. Endocrinol..

[70] Musso G., Gambino R., Cassader M. (2013). Cholesterol metabolism and the pathogenesis of non-alcoholic steatohepatitis. Prog. Lipid Res..

[71] Fernández-Suárez M.E., Daimiel L., Villa-Turégano G., Pavón M.V., Busto R., Escolà-Gil J.C., Platt F.M., Lasunción M.A., Martínez-Botas J., Gómez-Coronado D. (2021). Selective estrogen receptor modulators (SERMs) affect cholesterol homeostasis through the master regulators SREBP and LXR. Biomed. Pharmacother..

[72] Brown M.S., Goldstein J.L. (1997). The SREBP pathway: Regulation of cholesterol metabolism by proteolysis of a membrane-bound transcription factor. Cell.

[73] Chen M., Zhang J., Sampieri K., Clohessy J.G., Mendez L., Gonzalez-Billalabeitia E., Liu X.S., Lee Y.R., Fung J., Katon J.M. (2018). An aberrant SREBP-dependent lipogenic program promotes metastatic prostate cancer. Nat. Genet..

[74] Li X., Wu J.B., Li Q., Shigemura K., Chung L.W.K., Huang W.-C. (2016). SREBP-2 promotes stem cell-like properties and metastasis by transcriptional activation of c-Myc in prostate cancer. Oncotarget.

[75] Hubalek M., Brunner C., Matthä K., Marth C. (2010). Resistance to HER2-targeted therapy: Mechanisms of trastuzumab resistance and possible strategies to overcome unresponsiveness to treatment. Wien. Med. Wochenschr..

[76] Wahdan-Alaswad R., Liu B., Thor A.D. (2020). Targeted lapatinib anti-HER2/ErbB2 therapy resistance in breast cancer: Opportunities to overcome a difficult problem. Cancer Drug Resist..

[77] Wang Y.-C., Morrison G., Gillihan R., Guo J., Ward R.M., Fu X., Botero M.F., Healy N.A., Hilsenbeck S.G., Phillips G.L. (2011). Different mechanisms for resistance to trastuzumab versus lapatinib in HER2-positive breast cancers—Role of estrogen receptor and HER2 reactivation. Breast Cancer Res..

[78] D’Amato V., Raimondo L., Formisano L., Giuliano M., De Placido S., Rosa R., Bianco R. (2015). Mechanisms of lapatinib resistance in HER2-driven breast cancer. Cancer Treat. Rev..

[79] Sethunath V., Hu H., De Angelis C., Veeraraghavan J., Qin L., Wang N., Simon L.M., Wang T., Fu X., Nardone A. (2019). Targeting the Mevalonate Pathway to Overcome Acquired Anti-HER2 Treatment Resistance in Breast Cancer. Mol. Cancer Res..

[80] Dinavahi S.S., Chen Y.-C., Gowda R., Dhanyamraju P.K., Punnath K., Desai D., Berg A., Kimball S.R., Amin S., Yang J.-M. (2022). Targeting Protein Translation in Melanoma by Inhibiting EEF-2 Kinase Regulates Cholesterol Metabolism though SREBP2 to Inhibit Tumour Development. Int. J. Mol. Sci..

[81] Liang L., Liu Y., Wu X., Chen Y. (2023). Artesunate induces ferroptosis by inhibiting the nuclear localization of SREBP2 in myeloma cells. Int. J. Med. Sci..

[82] Muto C., Yachi R., Aoki Y., Koike T., Igarashi O., Kiyose C. (2013). Gamma-tocotrienol reduces the triacylglycerol level in rat primary hepatocytes through regulation of fatty acid metabolism. J. Clin. Biochem. Nutr..

[83] Krycer J.R., Phan L., Brown A.J. (2012). A key regulator of cholesterol homoeostasis, SREBP-2, can be targeted in prostate cancer cells with natural products. Biochem. J..

[84] Munkacsi A.B., Porto A.F., Sturley S.L. (2007). Niemann-Pick type C disease proteins: Orphan transporters or membrane rheostats?. Future Lipidol..

[85] Roney J.C., Li S., Farfel-Becker T., Huang N., Sun T., Xie Y., Cheng X.-T., Lin M.-Y., Platt F.M., Sheng Z.-H. (2021). Lipid-mediated motor-adaptor sequestration impairs axonal lysosome delivery leading to autophagic stress and dystrophy in Niemann-Pick type C. Dev. Cell.

[86] O’Neill K.I., Kuo L.-W., Williams M.M., Lind H., Crump L.S., Hammond N.G., Spoelstra N.S., Caino M.C., Richer J.K. (2022). NPC1 Confers Metabolic Flexibility in Triple Negative Breast Cancer. Cancers.

[87] Garver W.S., Jelinek D., Francis G.A., Murphy B.D. (2008). The Niemann-Pick C1 gene is downregulated by feedback inhibition of the SREBP pathway in human fibroblasts. J. Lipid Res..

[88] Zeng L., Liao H., Liu Y., Lee T.S., Zhu M., Wang X., Stemerman M.B., Zhu Y., Shyy J.Y. (2004). Sterol-responsive element-binding protein (SREBP) 2 down-regulates ATP-binding cassette transporter A1 in vascular endothelial cells: A novel role of SREBP in regulating cholesterol metabolism. J. Biol. Chem..

[89] Wilhelm L.P., Wendling C., Védie B., Kobayashi T., Chenard M.P., Tomasetto C., Drin G., Alpy F. (2017). STARD 3 mediates endoplasmic reticulum-to-endosome cholesterol transport at membrane contact sites. EMBO J..

[90] Meneses-Salas E., García-Melero A., Kanerva K., Blanco-Muñoz P., Morales-Paytuvi F., Bonjoch J., Casas J., Egert A., Beevi S.S., Jose J. (2020). Annexin A6 modulates TBC1D15/Rab7/StARD3 axis to control endosomal cholesterol export in NPC1 cells. Cell Mol. Life Sci..

[91] Asif K., Memeo L., Palazzolo S., Frión-Herrera Y., Parisi S., Caligiuri I., Canzonieri V., Granchi C., Tuccinardi T., Rizzolio F. (2021). STARD3: A Prospective Target for Cancer Therapy. Cancers.

[92] Skorda A., Lauridsen A.R., Wu C., Huang J., Mrackova M., Winther N.I., Jank V., Sztupinszki Z., Strauss R., Bilgin M. (2023). Activation of invasion by oncogenic reprogramming of cholesterol metabolism via increased NPC1 expression and macropinocytosis. Oncogene.

[93] Davis O.B., Shin H.R., Lim C.-Y., Wu E.Y., Kukurugya M., Maher C.F., Perera R.M., Ordonez M.P., Zoncu R. (2021). NPC1-mTORC1 Signaling Couples Cholesterol Sensing to Organelle Homeostasis and Is a Targetable Pathway in Niemann-Pick Type C. Dev. Cell.

[94] Nguyen M.K.L., Jose J., Wahba M., Bernaus-Esqué M., Hoy A.J., Enrich C., Rentero C., Grewal T. (2022). Linking Late Endosomal Cholesterol with Cancer Progression and Anticancer Drug Resistance. Int. J. Mol. Sci..

[95] Lyu J., Yang E.J., Shim J.S. (2019). Cholesterol Trafficking: An Emerging Therapeutic Target for Angiogenesis and Cancer. Cells.

[96] Oni T.E., Biffi G., Baker L.A., Hao Y., Tonelli C., Somerville T.D.D., Deschênes A., Belleau P., Hwang C.-I., Sánchez-Rivera F.J. (2020). SOAT1 promotes mevalonate pathway dependency in pancreatic cancer. J. Exp. Med..

[97] Da Dalt L., Pedrelli M., Pramfalk C., Norata G., Parini P. (2022). Cholesterol trapping by SOAT1 induces mitochondrial cholesterol accumulation and decrease oxidative metabolism. Atherosclerosis.

[98] Cheng X., Li J., Guo D. (2018). SCAP/SREBPs are Central Players in Lipid Metabolism and Novel Metabolic Targets in Cancer Therapy. Curr. Top. Med. Chem..

[99] Eckhardt C., Sbiera I., Krebs M., Sbiera S., Spahn M., Kneitz B., Joniau S., Fassnacht M., Kübler H., Weigand I. (2022). High expression of Sterol-O-Acyl transferase 1 (SOAT1), an enzyme involved in cholesterol metabolism, is associated with earlier biochemical recurrence in high risk prostate cancer. Prostate Cancer Prostatic Dis..

[100] Ohshiro T., Ohtawa M., Nagamitsu T., Matsuda D., Yagyu H., Davis M.A., Rudel L.L., Ishibashi S., Tomoda H. (2015). New pyripyropene A derivatives, highly SOAT2-selective inhibitors, improve hypercholesterolemia and atherosclerosis in atherogenic mouse models. J. Pharmacol. Exp. Ther..

[101] Smith D.C., Kroiss M., Kebebew E., Habra M.A., Chugh R., Schneider B.J., Fassnacht M., Jafarinasabian P., Ijzerman M.M., Lin V.H. (2020). A phase 1 study of nevanimibe HCl, a novel adrenal-specific sterol O-acyltransferase 1 (SOAT1) inhibitor, in adrenocortical carcinoma. Investig. New Drugs.

[102] Xu H., Xia H., Zhou S., Tang Q., Bi F. (2021). Cholesterol activates the Wnt/PCP-YAP signaling in SOAT1-targeted treatment of colon cancer. Cell Death Discov..

[103] Lye S.-H., Chahil J.K., Bagali P., Alex L., Vadivelu J., Ahmad W.A.W., Chan S.-P., Thong M.-K., Zain S.M., Mohamed R. (2013). Genetic polymorphisms in LDLR, APOB, PCSK9 and other lipid related genes associated with familial hypercholesterolemia in Malaysia. PLoS ONE.

[104] Wang L., Li S., Luo H., Lu Q., Yu S. (2022). PCSK9 promotes the progression and metastasis of colon cancer cells through regulation of EMT and PI3K/AKT signaling in tumor cells and phenotypic polarization of macrophages. J. Exp. Clin. Cancer Res..

[105] Kwon H.J., Lagace T.A., McNutt M.C., Horton J.D., Deisenhofer J. (2008). Molecular basis for LDL receptor recognition by PCSK9. Proc. Natl. Acad. Sci. USA.

[106] Chora J.R., Medeiros A.M., Alves A.C., Bourbon M. (2018). Analysis of publicly available LDLR, APOB, and PCSK9 variants associated with familial hypercholesterolemia: Application of ACMG guidelines and implications for familial hypercholesterolemia diagnosis. Genet. Med..

[107] Zia S., Batool S., Shahid R. (2020). Could PCSK9 be a new therapeutic target of Eugenol? In vitro and in silico evaluation of hypothesis. Med. Hypotheses.

[108] Abdelwahed K.S., Siddique A.B., Mohyeldin M.M., Qusa M.H., Goda A.A., Singh S.S., Ayoub N.M., King J.A., Jois S.D., El Sayed K.A. (2020). Pseurotin A as a novel suppressor of hormone dependent breast cancer progression and recurrence by inhibiting PCSK9 secretion and interaction with LDL receptor. Pharmacol. Res..

[109] Wong C.C., Wu J.-L., Ji F., Kang W., Bian X., Chen H., Chan L.-S., Luk S.T.Y., Tong S., Xu J. (2022). The cholesterol uptake regulator PCSK9 promotes and is a therapeutic target in APC/KRAS-mutant colorectal cancer. Nat. Commun..

[110] Burnett J.R., Hooper A.J. (2023). MK-0616: An oral PCSK9 inhibitor for hypercholesterolemia treatment. Expert Opin. Investig. Drugs.

[111] Liu C., Chen J., Chen H., Zhang T., He D., Luo Q., Chi J., Hong Z., Liao Y., Zhang S. (2022). PCSK9 Inhibition: From Current Advances to Evolving Future. Cells.

[112] Ballantyne C.M., Banka P., Mendez G., Garcia R., Rosenstock J., Rodgers A., Mendizabal G., Mitchel Y., Catapano A.L. (2023). Phase 2b Randomized Trial of the Oral PCSK9 Inhibitor MK-0616. J. Am. Coll. Cardiol..

[113] Lagunas-Rangel F.A. (2025). Cholesterol effects on the tumor immune microenvironment: From fundamental concepts to mechanisms and implications. Front. Oncol..

[114] Su Q., Yao J., Farooq M.A., Ajmal I., Duan Y., He C., Hu X., Jiang W. (2024). Modulating Cholesterol Metabolism via ACAT1 Knockdown Enhances Anti-B-Cell Lymphoma Activities of CD19-Specific Chimeric Antigen Receptor T Cells by Improving the Cell Activation and Proliferation. Cells.

[115] Li S., Zhang Y., Tong H., Sun H., Liao H., Li Q., Ma X. (2025). Metabolic regulation of immunity in the tumor microenvironment. Cell Rep..

[116] Liu X., Ren L., Shi R. (2025). Lipid Metabolic Reprogramming and the Tumor Immune Microenvironment: A New Strategy for Early Diagnosis and Cancer Prevention. Cancer Screen. Prev..

[117] Plata-Gomez A.B., Chen W., Ho P.C., Ling G.S. (2025). Mitochondrial lipid metabolism in tumor immunosurveillance and evasion. Trends Immunol..

[118] Zhao L., Zheng R., Liu W., Li X., Liu H., Yin H., Zhang R., Yang A. (2025). Cholesterol metabolism: A new checkpoint in cancer immunity. Trends Mol. Med..

[119] Padyana A.K., Gross S., Jin L., Cianchetta G., Narayanaswamy R., Wang F., Wang R., Fang C., Lv X., Biller S.A. (2019). Structure and inhibition mechanism of the catalytic domain of human squalene epoxidase. Nat. Commun..

[120] Ohmoto T., Nishitsuji K., Yoshitani N., Mizuguchi M., Yanagisawa Y., Saito H., Sakashita N. (2015). K604, a specific acyl-CoA:cholesterol acyltransferase 1 inhibitor, suppresses proliferation of U251-MG glioblastoma cells. Mol. Med. Rep..

[121] Guo D., Reinitz F., Youssef M., Hong C., Nathanson D., Akhavan D., Kuga D., Amzajerdi A.N., Soto H., Zhu S. (2011). An LXR agonist promotes glioblastoma cell death through inhibition of an EGFR/AKT/SREBP-1/LDLR-dependent pathway. Cancer Discov..

[122] Gambhire V.M., Salunkhe S.M., Gambhire M.S. (2018). Atorvastatin-loaded lipid nanoparticles: Antitumor activity studies on MCF-7 breast cancer cells. Drug Dev. Ind. Pharm..

[123] Wang R., Gilbert C., Tahiri H., Yang C., Landreville S., Hardy P. (2025). MiR-181a-driven downregulation of cholesterol biosynthesis through SREBP2 inhibition suppresses uveal melanoma metastasis. J. Exp. Clin. Cancer Res..

[124] Xie X., Wang W., Zhang H., Zhao S., Zhang N., Gao Y., Liu Q., Chen X. (2025). Cholesterol-induced colorectal cancer progression and its mitigation through gut microbiota remodeling and simvastatin treatment. BMC Cancer.

